# High-Density Genetic Map Construction and QTL Mapping of Leaf and Needling Traits in *Ziziphus jujuba* Mill

**DOI:** 10.3389/fpls.2019.01424

**Published:** 2019-11-22

**Authors:** Zhongtang Wang, Zhong Zhang, Haixia Tang, Qiong Zhang, Guangfang Zhou, Xingang Li

**Affiliations:** ^1^College of Forestry, Northwest A&F University, Yangling, China; ^2^Country Shandong Institute of Pomology, Taian, China; ^3^Research Centre for Jujube Engineering and Technology of State Forestry and Grassland Administration, Northwest A&F University, Yangling, China; ^4^Key Comprehensive Laboratory of Forestry of Shaanxi Province, Northwest A&F University, Yangling, China

**Keywords:** jujube (*Ziziphus jujuba Mill*.), GBS (genotyping by sequencing), genetic map, leaf and needling traits, QTL

## Abstract

The Chinese jujube (*Ziziphus jujuba* Mill., 2*n* = 2*x* = 24), one of the most popular fruit trees in Asia, is widely cultivated and utilized in China, where it is traditionally consumed as both a fresh and dried food resource. A high-density genetic map can provide the necessary framework for quantitative trait loci (QTL) analyses and map-based gene cloning and molecular breeding. In this study, we constructed a new high-density genetic linkage map *via* a genotyping-by-sequencing approach. For the consensus linkage map, a total of 3,792 markers spanning 2,167.5 cM were mapped onto 12 linkage groups, with an average marker interval distance of 0.358 cM. The genetic map anchored 301 Mb (85.7%) of scaffolds from the sequenced *Z*. *jujuba* “Junzao” genome. Based on this genetic map, 30 potential QTLs were detected, including 27 QTLs for leaf traits and 3 QTLs for needling length. This high-density genetic map and the identified QTLs for relevant agronomic traits lay the groundwork for functional genetic mapping, map-based cloning, and marker-assisted selection in jujube.

## Introduction

Chinese jujube (*Ziziphus jujuba* Mill., 2*n* = 2*x* = 24) is a popular fruit tree in Asia. The fruit crop has been widely cultivated across Northern China for 7,000 years (Qu and Wang, 1993; Liu and Wang, 2008), and nowadays there are more than 840 cultivars (Liu et al., 2014a). These cultivars are mostly landraces which have not been subjected to modern breeding. The jujube is domesticated from its wild relatives, *Z. jujuba* Mill. var. *spinosa* (Bunge) Hu ex H. F. Chow, and this long process has been suggested to be linked with human selection and natural reproduction (Liu and Wang, 2008). The wild jujube is a small shrub, typically possessing thorny branches and ovate-acute leaves with three conspicuous veins at the base and finely toothed margins (Gupta, 2004). They can withstand extreme arid conditions and produce reasonable yields. In contrast, the Chinese jujube has become greatly differentiated during the long history of evolution (Liu, 2010). Chinese jujube has diverse stipular spines (strong, weak, and absent) and leaf shape (oval, ovoid in shape, ovate-lanceolate). Importantly, leaf traits can influence the fitness of trees through biochemical, physiological, morphological, and developmental mechanisms (Donovan et al., 2011). Leaves are also the major organ of photosynthesis in plants, and photosynthesis of jujube is highly sensitive to water deficit, directly affecting development and productivity (Cui et al., 2009). An additional morphological feature of jujube is needling, which causes inconveniences in field operation of farmers and may even cause injury (Qi et al., 2009). Thus, mapping of genes controlling leaf and needling traits and development of applicable markers, are of significant value in jujube farming.

In the past 15 years, substantial progress has been made towards the development of genetic markers and construction of linkage maps in jujube (**Supplementary Table S6**). The first genetic map is based on the F_1_ progenies from the cross between “Dongzao” and “Linyillizao,” with 128 AFLP markers, consisting of seven linkage groups (LGs), and spanning 458.66 cM (Lu et al., 2005). The map was then further developed by RAPD and SSR markers, with the marker numbers ranging from 333 to 423 by Shen (2005), Qi (2009), and Xu (2012). However, the map is limited by low marker density, meaning it is often unsuitable for breeding purposes. More recently, the jujube reference genome sequences have been released (Liu et al., 2014b; Huang et al., 2016), making it possible to develop more genetic markers for jujube. With the development of next generation sequencing technologies, two sets of 2,872 and 2,540 single nucleotide polymorphisms (SNPs) were identified from two F_1_ populations, “Dongzao” × “JMS2” and “Dongzao” × “Zhongningyunzao,” respectively. These reports demonstrate a robust and powerful approach for genotyping in jujube using Illumina sequencing technology (Zhao et al., 2014; Zhang et al., 2016). However, there is still no saturated genetic map for QTL localization in Chinese jujube.

Genotyping by sequencing (GBS) identifies SNPs within restriction-site-associated DNA sequences at many loci throughout the genome (Miller et al., 2007). A combination of three restriction enzymes with distinct restriction sites, *MseI* (TTAA), *HaeIII* (GGCC), and *EcoRI* (GAATTC), have been previously used for GBS library construction. This approach increased the tag number, sequencing depth, and genome coverage, while also providing additional opportunities to detect suitable regions for targeted fragments (Carlson et al., 2015). Recent studies have shown that GBS is an efficient and low-cost approach for SNP marker development in jujube (Zhang et al., 2016; Wu et al., 2017). Thus, we sought to use a GBS strategy to construct a new high-density genetic map for jujube.

In this study, we generated a new high-density genetic map with an F_1_ population crossed by “Dongzao” × “Jinsi4.” With this genetic map, we also identified genomic regions that were associated with important horticultural traits, such as leaf length, leaf width, leaf area, leaf shape index, specific leaf weight, chlorophyll content, and needling length.

## Materials and Methods

### Mapping Population and DNA Isolation

The F_1_ population of 103 progenies generated from “Jinsi4” (JS) × “Dongzao” (DZ) in the jujube garden of Shandong Institute of Pomology in Taian, Shandong, China (N36.21°, E117.16°), was used to make a high-density genetic map (Wang et al., 2019).

The leaf samples were collected at four weeks after sprouting from each F_1_ individual and the parents for DNA isolation in May 2016. Collected samples were immediately frozen in liquid nitrogen and transferred to −80°C. Approximately 200 mg of each sample was ground in liquid nitrogen for genomic DNA isolation using a plant genomic DNA extraction kit 9768 (Takara, Dalian, China) following the manufacturer’s protocol. A NanoDrop 2000 UV-Vis spectrophotometer (Thermo Fisher Scientific, USA) was used to determine the DNA concentration in each sample.

### GBS and High-Throughput Sequencing

A GBS strategy was used to develop SNP markers as previously described (Zhang et al., 2016). Briefly, approximately 0.1 to 1 µg of genomic DNA was incubated at 37°C with *MseI* (New England Biolabs, NEB), T4 DNA ligase (New England Biolabs, NEB), ATP (New England Biolabs, NEB), and an *MseI* Y adapter N containing barcodes, and the samples were then heat-inactivated at 65°C. Two additional enzymes, *HaeIII* and *EcoRI* (New England Biolabs, NEB), were simultaneously added into the *MseI* digestions to further digest the fragments at 37°C. Then, the digested fragments with ligations were purified with Agencourt AMPure XP beads (Beckman Coulter, Inc.) and subjected to PCR amplification with the Phusion Master Mix (New England Biolabs, NEB) using both universal primers as well as i5 and i7 index primers (Illumina). The PCR products were purified using Agencourt AMPure XP beads (Beckman Coulter, Inc.), pooled, and separated by electrophoresis on a 2% agarose gel. Fragments of 400 to 450 bp (with indexes and adaptors) were excised from the gel and purified using a gel extraction kit (QIAGEN). These purified products were further cleaned using Agencourt AMPure XP beads (Beckman Coulter, Inc.) prior to sequencing. Then, paired-end 150 bp sequencing was performed on the selected tags on the Illumina HiSeq 4000 platform.

### Data Analysis

To ensure that sequencing reads were reliable and without artificial bias raw data in the Fastq format was initially processed through a series of quality control (QC) procedures using in-house C scripts. QC standards were as follows: (1) reads with ≥10% unidentified nucleotides (N) were removed; (2) reads with >50% bases having a phred quality <5 were removed; (3) reads with >10 nt aligned to the adapter, allowing ≤10% mismatches were removed; (4) reads containing the *Haell* or *EcoRI* enzyme sequence were removed. BWA (Burrows-Wheeler Aligner) (Li and Durbin, 2009) was used to align the clean reads of each sample to the reference genome (settings: mem-t 4-k 32-M-R). Alignment files were converted to BAM files using the SAMtools software (Li et al., 2009) (settings: –bS –t). If multiple read pairs had identical external coordinates, only the pair with the highest mapping quality was retained.

### SNP Calling and Genotyping

Variant calling was performed for all samples using the GATK software (Mckenna et al., 2010). UnifiedGenotyper was used to estimate genotype and gene frequencies. Unreliable SNPs were eliminated *via* a filtering process. SNP calling was performed for both parents and progenies using the SAMtools software (Li et al., 2009). SNPs were filtered using a house-in Perl script. Polymorphic markers between the two parents were detected and classified into eight segregation patterns (ab × cd, ef × eg, hk × hk, lm × ll, nn × np, aa × bb, ab × cc, and cc × ab) according to the CP model in JoinMap 4.1 software (Van Ooijen and Voorrips, 2001). For the F_1_ population, markers with the genotypes of hk × hk, lm × ll, and nn × np were chosen for genetic mapping.

### Map Construction and Anchoring Sequence Scaffolds

Prior to map construction, SNP markers were further filtered using the parameters of segregation distortion (*p* < 0.01), integrity (> 95%), or the presence of abnormal bases with a house-in script. Then, the filtered SNP markers were sorted using the maximum-likelihood method and corrected by Smooth algorithms in JoinMap4.1. The Kosambi mapping function was then used to calculate marker distances (Zhang et al., 2016). The integrated maps for both the male and female parents were computed using the combined group for map integration function in the MergeMap software (Wu et al., 2008). A Perl script SVG was used to visualize the exported maps. The number and linkage distance of gaps representing the interval between two markers on 12 LGs were counted.

### Anchoring Sequenced Scaffolds to the Genetic Map

For the collinearity analysis, markers localized on the genetic map were anchored with the assembled scaffolds of jujube genome (NCBI accession: LPXJ00000000) (Huang et al., 2016) using a Perl script. The mapping results were further visualized by joining the LG and anchored scaffolds together with grey lines (Zhang et al., 2016).

### Phenotyping Traits

All measurements were performed on the 103 progenies of JS × DZ. The needles and leaf traits were investigated in May and July 2016, 2017, and 2018. Fifteen to 20 leaves were picked from middle shedding shoots of the jujube on each tree, and six major leaf traits were measured. These traits were leaf length, leaf width, leaf area, leaf shape index, specific leaf weight, and chlorophyll content. Ten to 20 needles were picked from the biennial shoots. Leaf length, leaf width, and needle length were measured using Vernier calipers, and leaf area was measured using a Li-3000c Leaf Area Meter (Li-cor Inc., USA). Chlorophyll content and leaf weight was measured by a SPAD-502 chlorophyll meter (Zhejiang Top Instrument Co., Ltd.) and leaf weight was measured using an analytical balance (Zhejiang Top Instrument Co., Ltd.). All of the measurements were repeated three times. Reported phenotypic data were the average of three years, and were analyzed using the SPSS v18.0 statistical software package (Zhang et al., 2016).

### QTL Analysis

Seven phenotypic traits were subjected to QTL analysis using the MQM mapping method of the MapQTL software (Van Ooijen, 2009). A 1,000 permutation test at a 95% confidence level was used to determine the LOD thresholds (use random genotypes to associate with phenotypes, take Max in every time permutation), with significance set at *p* < 0.05 (Van Ooijen, 1999). After 1,000 permutation test, a LOD threshold of 3.0 was set to identify significant QTLs at the 95% confidence level. Ranges above the LOD threshold of 3.0 were identified as QTL intervals. Markers located at or flanking the peak LOD value of a QTL were identified as QTL associated markers (Sun et al., 2015).

## Results

### Quality Evaluation of Sequencing Data

A total of 40.31 Gb of clean reads (99.99% of total raw reads) were generated by sequencing the parents and 103 progenies. After data filtering, 99.99% of reads were of high quality, with an average Q20 ratio of 99.9% and a GC content of 35.94%. The parents were sequenced at a higher depth to enhance the chances of SNP detection. Finally, clean data covering 1,592,370,720 bp (99.99%) and 1,466,831,520 bp (99.99%) were obtained for the female and male parents, respectively. For each individual plant, the clean data ranged from 203,802,048 to 618,848,928 bp in coverage, with an average of 361,682,155 bp. The average number of total reads for parents and progenies were 1,529,724,096 and 361,713,312 bp, respectively (**Supplementary Table S1**).

Paired-end reads of clean data for the two parents and the F_1_ progenies were computed using the BWA Comparison software (parameters: mem-t 4-k 32-M-R). Comparison results were processed with SAMtools for format conversion. The published jujube genome has 351,295,593 bp. High-quality clean reads were aligned to this genome. In our study, 11,058,130 and 10,186,330 aligned clean reads were obtained for the female and male parents, respectively. For F_1_ individuals, an average of 2,511,682 clean reads was aligned to the reference genome. Mapping rates of the 103 F_1_ individuals were between 96.6% and 98.15% using the Perl script (**Supplementary Table S2**).

### SNP Calling and Genotyping

In total, 378,500 and 346,669 SNPs were detected in the female and male parents, respectively. For F_1_ individuals, an average of 224,432 SNPs were discovered for each progeny. The parents exhibited a lower SNP heterozygosity rate (39.96%) than F_1_ individuals (41.45%) (**Supplementary Table S3**).

**Figure 1:**
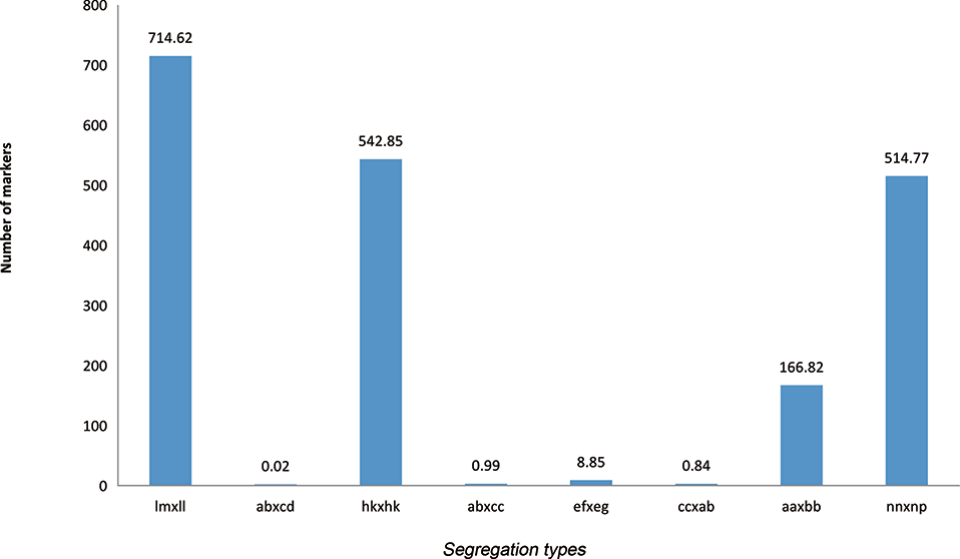
Segregation types of polymorphic SNP markers. The *x* axis indicates the eight segregation types; the *y* axis indicates the corresponding number of markers.

By excluding missing information, a combined 194,976 polymorphic SNPs were detected between the two parents. These SNPs were classified into eight segregation types according to the CP model using JoinMap 4.1. Among eight detected marker patterns, four major patterns including lm × ll, aa × bb, hk × hk, and nn × np accounted for nearly 99.5%, while the other four patterns, ab × cd, ab × cc, cc × ab, and ef × eg, only accounted for 0.5%. Only segregation types lm × ll, nn × np, and hk × hk were selected for genotyping in F_1_ individuals. The final number of available markers was 177,224 (**Figure 1**).

### Genetic Linkage Map Construction

Prior to map construction, we generated 35,509 candidate markers by further filtering the abnormal bases and a rate lower than 95% integrity in each individual. By screening the threshold of segregation distortion (*p* < 0.01), 22,424 markers were used for the final map construction (**Table 1**).

**Table 1:** Basic features of the 12 linkage groups (LGs) in the high-density genetic map generated from Dongzao × Jinsi4.

**Chr**	Number of markers (without consistent loci)			Number of markers (including consistency loci)			Size (cM)			Average distance between adjacent markers (cM)			Maximum distance between markers (cM)			Number of gaps <br/><5 cM			Ratio of gaps <5 cM (%)		
**F- M**	**M-M**	**I-M**	**F- M**	**M-M**	**I-M**	**F- M**	**M-M**	**I-M**	**F- M**	**M-M**	**I-M**	**F- M**	**M-M**	**I-M**	**F- M**	**M-M**	**I-M**	**F- M**	**M-M**	**I-M**
LG1	405	356	616	1,418	1,016	2,026	197.23	206.957	204.084	0.49	0.58	0.33	9.657	9.446	5.457	400	350	614	99	99	100
LG2	160	139	239	951	616	1,345	67.281	98.307	84.075	0.42	0.71	0.35	4.801	41.403	6.092	159	135	237	100	98	100
LG3	313	296	480	1,154	966	1,745	203.896	176.471	193.277	0.65	0.6	0.4	7.813	8.441	4.557	308	291	479	99	99	100
LG4	281	262	449	1,072	877	1,648	134.01	117.571	137.332	0.48	0.45	0.31	9.416	7.304	7.305	276	257	447	99	98	100
LG5	169	259	319	673	857	1,228	48.508	99.655	75.062	0.29	0.38	0.24	5.838	4.988	2.942	167	258	318	99	100	100
LG6	519	479	762	1,674	1,511	2,504	259.068	282.458	275.955	0.5	0.59	0.36	6.194	6.974	4.689	512	470	761	99	98	100
LG7	45	236	237	141	963	969	3.922	109.469	57.683	0.09	0.46	0.24	0.981	5.106	2.553	44	234	236	100	100	100
LG8	446	333	627	1,652	1,067	2,213	340.729	258.691	300.919	0.76	0.78	0.48	24.62	10	14.822	431	319	616	97	96	98
LG9	305	251	463	1,256	825	1,767	165.852	215.449	194.507	0.54	0.86	0.42	13.403	47.08	9.954	300	242	457	99	97	99
LG10	378	249	476	1,355	1,001	1,771	193.719	193.323	195.995	0.51	0.78	0.41	8.47	28.896	6.071	372	2,441	470	99	97	99
LG11	517	243	672	2,492	779	3,005	333.598	300.283	318.495	0.65	1.24	0.47	12.014	26.952	5.887	513	231	669	99	95	100
LG12	312	233	446	1,701	945	2,203	145.246	117.009	131.127	0.47	0.5	0.29	6.182	14.488	3.575	310	229	445	100	99	100
Total	3,850	3,336	3,792	15,539	11,423	22,424	2,093.059	2,175.643	2,167.511	—	—	—	—	—	—	3,792	5,457	5,749	—	—	—

F-M, female map; M-M, male map; I-M, integrated map; number of gaps <5 cM, number of genetic distances between markers less than 5cM; ratio of gaps <5 cM, number of gaps with a genetic distance of less than 5 cM between marks as a proportion of the total number of marks.

In total, 11,423 (3,336 without consistent loci) markers and 15,539 (3,850 without consistent loci) markers fell into 12 LGs on the male and female maps, respectively. The genetic lengths were 2,175.643 and 2,093.059 cM, with average marker intervals of 0.66 and 0.4875 cM (**Supplementary Tables S4** and **S5**, **Supplementary Figures S1** and **S2**).

**Figure 2:**
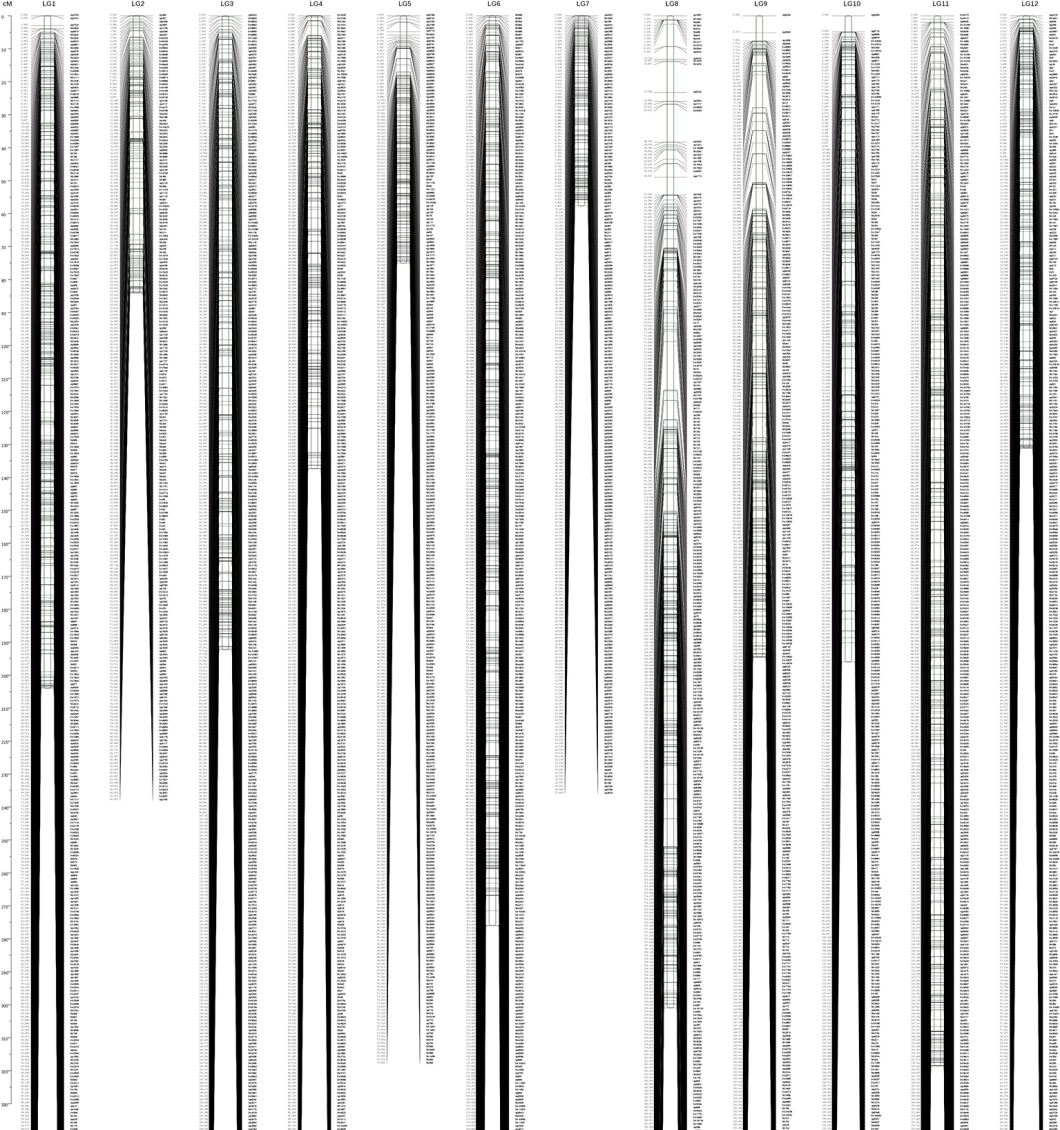
Integrated genetic map with 12 linkage groups. The *x* axis indicates the numbers of linkage groups; the *y* axis indicates the genetic length (cM).

The combined map spanned 2,167.511 cM with 22,424 (3,792 without consistent loci) markers falling into 12 LGs, with an average marker interval of 0.358 cM and average chromosome length of 180.63 cM (**Figure 2**). Among the 12 LGs, LG11 was the largest group, with a genetic distance of 318.5 cM and 3,005 markers (**Table 2**). LG07 was the shortest group with 969 markers and spanning 57.68 cM. The average marker interval ranged from 0.24 to 0.48 cM, with an average distance of 0.358 cM (**Table 1**). Among these markers, 5,774 gaps were detected. Of these, 5,749 gaps (99.6%) were less than 5 cM, 23 gaps were between 5 and 10 cM, and only two gaps were larger than 10 cM, which were on LG08.

**Table 2:** Anchored sequenced genome scaffolds of *Z. jujuba* Junzao with SNP markers.

Linkage group (LG)	Number of scaffolds	Number of anchored markers	Genetic length (cM)	Physical length (Kb)	Relationship genetic map to physical length (Kb/cM)
LG1	73	616	204.08	41,585.24	203.77
LG2	67	239	84.08	25,335.53	301.34
LG3	83	480	192.28	18,768.06	97.61
LG4	26	449	137.33	16,610.93	120.95
LG5	45	319	75.06	14,103.02	187.88
LG6	94	762	275.96	22,652.56	82.09
LG7	49	237	57.68	13,694.57	237.41
LG8	89	627	300.92	16,671.51	55.40
LG9	47	463	194.51	10,190.79	52.39
LG10	81	476	196.00	40,322.86	205.73
LG11	100	672	318.50	46,886.24	147.21
LG12	98	446	131.13	34,754.20	265.04
Average	71	482	180.63	25,131.29	163.07
Total	852	5,786	2,167.51	301,575.52	1,956.84

### Anchoring Scaffolds of the Sequenced Jujube Genome to the Genetic Map

**Figure 3:**
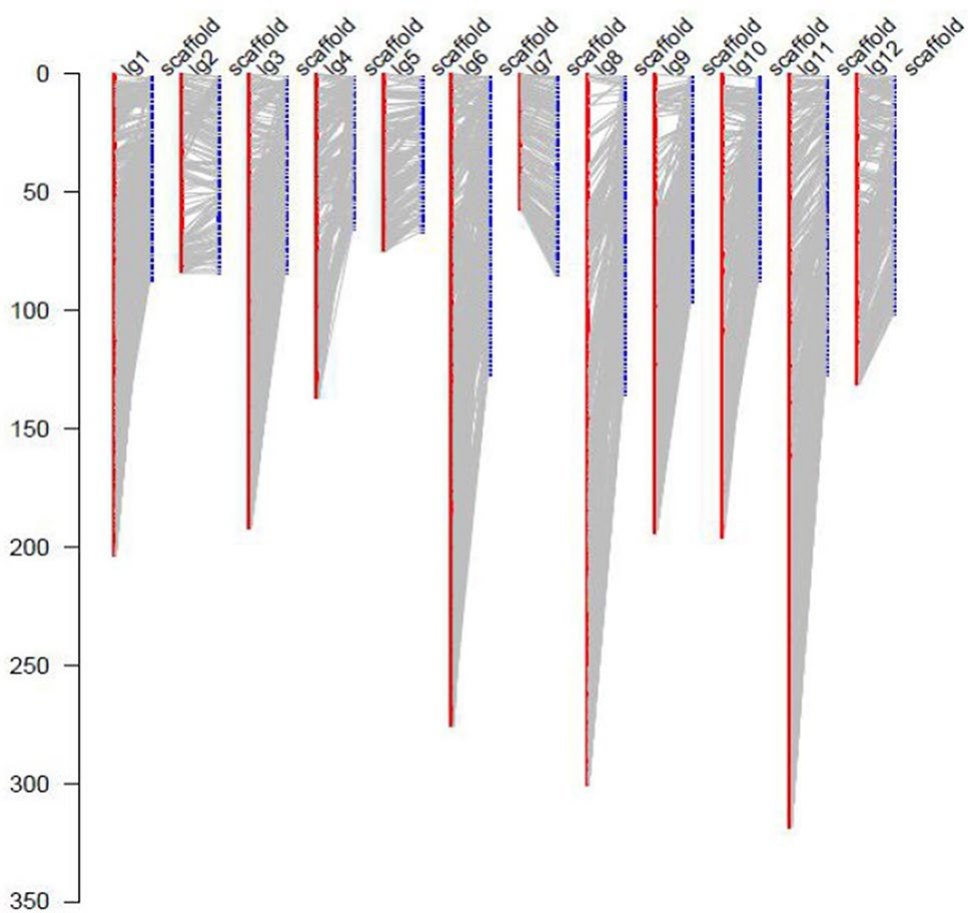
Anchoring of genome assembled scaffolds (LPXJ00000000) to the 12 linkage groups. The *x* axis indicates linkage groups (LG1–12) and scaffolds, the *y* axis indicates the genetic length (cM).

For the collinearity analysis, 5,786 markers (without consistent loci) localized on the genetic map of JS × DZ were anchored with the assembled scaffolds of jujube genome. In total, 852 unique scaffolds representing 301 Mb (85.7%) of the total genome were localized on the 12 LGs (**Figure 3**). The sequenced jujube genome (“Junzao”) consisted of 36,147 scaffolds covering 351 Mb of sequences. LG11 anchored the highest number of scaffolds with a physical length of 46.9 Mb. LG4 anchored the lowest number of scaffolds with a length of 16.6 Mb. The correlation between genetic and physical length was 163.07 Kb/cM on average (**Table 2**).

### Variations of F_1_-Generation Leaf and Needling Characteristics

The morphological characteristics of leaf and needling in F_1_-generation were measured (**Supplementary Tables S7** and **S8**). The differences between leaf and needling parameters among F_1_ individuals were all extremely significant (*p* < 0.0001). The coefficients of variation for the progeny leaf features were all lower than 30%. In particular, the variation in leaf area ranged from 3.69 to 19.08 cm^2^, and its coefficient of variation reached up to 27.75%. The variation in leaf shape index ranged from 1.45 to 2.66, and the coefficient of variation was 12.34%. The average specific leaf weight of F_1_-generation leaves was 49.39, with a variation range from 27.29 to 107.78. The average chlorophyll content of F_1_-generation leaves was 17.46 SPAD, which was close to that of parents (18.02 SPAD). The variation coefficient of needling was 44.98%, the variation in needling length ranged from 2.47 to 37.2 cm.

### Normality Test of Leaf and Needling Characteristics

We tested the normality of leaf length, leaf width, leaf area, leaf shape index, specific leaf weight, chlorophyll content, and needling length (**Supplementary Table S8**). Based on Shapiro-Wilk tests, the W values of these parameters were 0.9913, 0.9829, 0.9828, 0.9873, 0.9825, 0.9838, and 0.9829. The P values were 0.7561, 0.2201, 0.2196, 0.4585, 0.2053, 0.2606, and 0.2252, respectively, which were all higher than the level of significance (*p* < 0.05). These results suggest the determined characters for F_1_-generation leaves and needlings conformed to a normal distribution (**Supplementary Figure S3**).

### QTL Analysis

**Figure 4:**
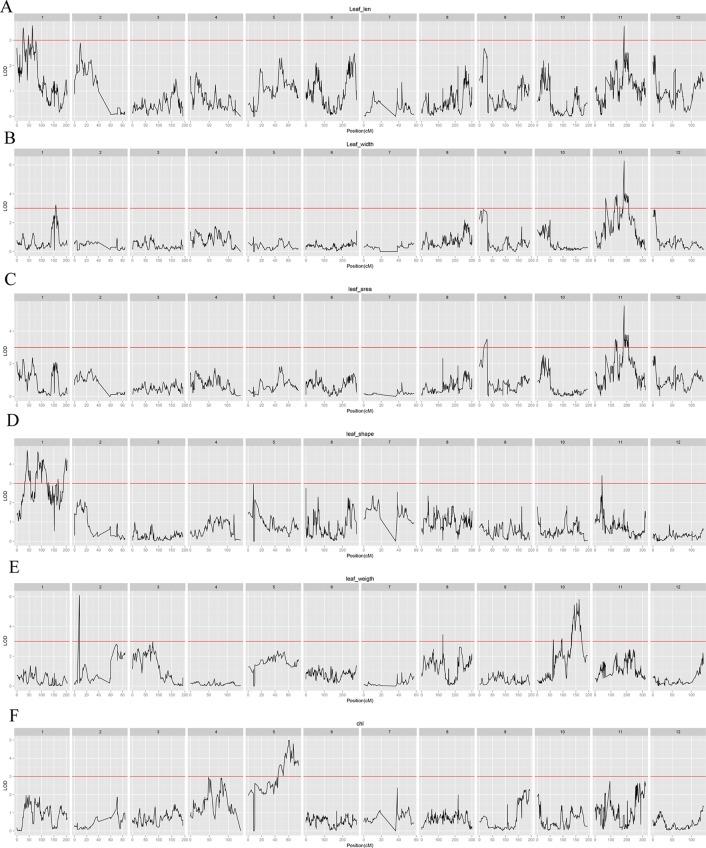
Leaf characteristic–associated QTLs in jujube among all linkage groups. **(A)**: Leaf length; **(B)**: Leaf width; **(C)**: Leaf area; **(D)**: Leaf shape index; **(E)**: Specific leaf weight; **(F)**: Chlorophyll content.

**Figure 5:**
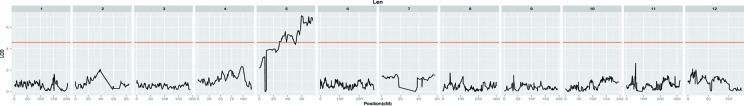
Needling length traits-associated QTLs in jujube among all linkage groups.

A total of 30 QTLs distributed across seven LGs were discovered by QTL analysis using our high-density map, with a range of two to seven QTLs for each trait. LOD scores for the seven qualitative traits ranged from 3.04 to 7.08. Twenty-seven QTLs were detected for leaf characteristics, including four QTLs for leaf length on LG1 and LG11; five QTLs for leaf width on LG1 and LG11; four QTLs for leaf area on LG9 and LG11; seven QTLs for leaf shape index on LG1 and LG11; five QTLs for specific leaf weight on LG2, LG8, and LG10; and two QTLs for chlorophyll content on LG5. In addition, three QTLs for needling length on LG5 were also detected by QTL. Interestingly, we found a co-localization of marker lm8132 for leaf length, leaf width, and leaf area (LL11.1, LW11.4 and LA11.3), and marker lm5435 for leaf width and leaf area (LW11.2 and LA11.2). The proportion of phenotypic variation ranged from 13.3% to 29.9%, with an average of 17.72%. Information related the detected QTLs for leaf and needling traits are shown in **Table 3**, **Figure 4** and **5** .

**Table 3:** Detected QTLs for leaf and needling traits.

Trait	Name	LG	Confidence interval	Peak position(cM)	Marker	LOD	Expl (%)	Left marker	Right marker
Leaf length	LL1.1	LG1	24.652-28.643	26.643	hk2424	3.49	14.8	np5687	lm7961
	LL 1.2	LG1	47.187-47.478	47.187	np3413	3.19	13.7	np1351	np1348
	LL 1.3	LG1	63.031-63.835	63.031	np4833	3.57	15.2	lm7964	np4834
	LL 11.1	LG11	183.456	183.456	lm8132	3.55	15.1	lm8142	lm8097
Leaf width	LW 1.1	LG1	156.968-158.451	158.212	lm841	3.23	13.8	lm827	np4117
	LW 11.1	LG11	66.276	66.276	lm4592	3.7	15.7	lm4603	lm4397
	LW 11.2	LG11	125.207-141.731	137.288	lm5435	3.93	16.5	lm5453	lm5443
	LW 11.3	LG11	155.94	155.94	lm9435	3.1	13.3	lm9417	lm9482
	LW 11.4	LG11	155.516-211.554	183.456	lm8132	6.28	25.1	lm8142	lm8097
Leaf area	LA 9.1	LG9	17.897-29.586	29.396	lm10895	3.5	15	lm10901	lm10859
	LA 11.1	LG11	127.953-131.714	130.193	lm8112	3.5	15	lm4369	np6092
	LA 11.2	LG11	131.743-137.89	137.288	lm5435	3.41	14.7	lm5453	lm5443
	LA 11.3	LG11	177.593-211.5454	183.456	lm8132	5.55	22.7	lm8142	lm8097
Leaf shape index	LSI 1.1	LG1	32.359-45.507	42.522	hk1038	4.7	19.6	np1345	np1393
	LSI 1.2	LG1	76.009-99.771	84.904	np1399	4.64	19.4	np1398	np1400
	LSI 1.3	LG1	100.704-122.92	107.141	hk240	4.25	17.9	lm2016	lm2014
	LSI 1.4	LG1	122.951-128.967	128.967	np1441	3.04	13.2	lm620	np1446
	LSI 1.5	LG1	166.782-166.788	166.782	np4109	3.22	13.9	np4110	np2845
	LSI 1.6	LG1	190.589-204.084	198.951	np6460	4.3	18.1	np274	np6464
	LSI 11.1	LG11	43.104	43.104	lm1922	3.41	14.7	lm1910	lm6531
Specific leaf weight	SLW2.1	LG2	8.08	8.08	lm6445	6.08	24.6	lm6688	lm4156
	SLW8.1	LG8	126.612	126.612	lm3029	3.46	14.9	lm3049	lm2999
	SLW10.1	LG10	62.794	62.794	lm9078	3.11	13.5	lm9086	lm9075
	SLW10.2	LG10	93.959-96.141	96.141	hk4002	3.19	13.8	hk3989	lm10062
	SLW10.3	LG10	132.888-172.407	162.643	np3953	5.81	23.7	np3950	np5078
Chlorophyll content	CC 5.1	LG5	44.6-52.156	47.55	hk2565	3.65	15.5	hk3190	hk650
	CC 5.2	LG5	53.875-75.062	60.827	hk2042	4.99	20.5	np4049	hk2040
Needling length	NL 5.1	LG5	28.625-38.398	36.735	np939	5.61	24.5	lm1786	np2959
	NL 5.2	LG5	38.696-52.156	40.674	hk2095	5.32	23.4	np2981	hk2100
	NL 5.3	LG5	53.873-75.062	59.359	lm10078	7.08	29.9	lm10069	lm10090

LG, linkage group.

## Discussion

### Construction of the Map Population

Construction of jujube mapping population is challenging, because jujube is a self-incompatible plant with a small flower size and high seedless percentage (Lu et al., 2005). Therefore, it is difficult to generate an F_1_ population. With the development of combination of SSR molecular identification and controlled pollination, a genetic mapping population was successfully constructed (Liu et al., 2008; Liu et al., 2014a). A previous study revealed that “Dongzao” and “Jinsixiaozao” had a distant genetic relationship (Huang et al., 2015), highlighting the potential for detecting more polymorphic markers in the derived population from the cross between them. Therefore, we constructed the jujube mapping population of “Dongzao” and “Jinsixiaozao 4,” expecting to generate a higher density genetic map than that currently available map by “Dongzao”× “Zhongningyuanzao.”

### Construction of a Genetic Map

SNP markers are an excellent tool for carrying out gene mapping experiments, as they are highly abundant and can be genotyped on a large scale (Slate et al., 2010). GBS utilizes one or multiple restriction enzymes to digest genomic DNA into fragments that can be sequenced on high-throughput platforms (Elshire et al., 2011). Multiplexing samples following the addition of unique barcodes avoids prohibitive sequencing costs (Pootakham et al., 2015). GBS has been used to construct genetic maps for many other species, such as wheat (Poland et al., 2012), bean (Schröder et al., 2016), rice (Leon et al., 2016), clam (Nie et al., 2017), grape (Wang et al., 2012), pear (Wu et al., 2014), peach (Bielenberg et al., 2015), and sweet cherry (Guajardo et al., 2015). It has also been used to construct maps for jujube (Zhang et al., 2016). In our study, a total of 40,315,919,328 bp of raw sequencing data with 99.9% clean data were generated, and 97.8% of the cleaned data were mapped to unique positions on the reference genome (Huang et al., 2016). Our data provide further evidence that GBS is a low cost, high efficiency, and rapid approach for SNP development and map construction.

We constructed a high-density genetic map for jujube using GBS technology to develop SNPs based on an F1 population. The integrated genetic linkage map comprised 3,792 SNP markers and spanned 2,167.511 cM, with an average marker interval of 0.358 cM. This map was divided into 12 LGs, which was consistent with the haploid chromosome number. Compared with previously reported linkage maps (Qi et al., 2009; Zhao et al., 2014; Zhang et al., 2016), we achieved a higher map density as well as shorter marker distances (0.358 cM). In addition, the mean interval distance is comparable to the currently reported high-density map with an interval distance of 0.34 cM crossed between “JMS2” and “Xing16” (Zhao et al., 2014). Therefore, our study demonstrates a highly efficient for map construction using the hybridization between two jujube cultivars with a distant genetic background.

High-density genetic linkage maps can facilitate genome assembly, and has been one of the fundamental components of genome sequencing (Gaur et al., 2012). Assisted by the high-density genetic map, we anchored 85% of the assembled scaffolds (310 Mb) of the jujube genome, higher than the genetic map of “Dongzao”× “Zhongningyuanzao” (Zhang et al., 2016). Comparison of our map to the previously anchored genome revealed a highly collinear relationship between genetic and physical maps. The inconsistent scaffold placement order could be explained by differences in the cultivars sequenced for the genetic map and genome sequencing. Rearrangements, translocations, gains or losses of DNA segments, and copy number variations have been widely observed in different genotypes of the same species (Swanson-Wagner et al., 2010; Zmienko et al., 2014). Alternatively, markers on different genetic maps could influence the anchoring results. Certainly, improper mapping or errors present in the genome assembly would also contribute to inconsistent placement orders (Soto et al., 2015). We calculated the relationships between the genetic and physical maps in the present study, finding that the ratio of physical/genetic distance was an average of 144.74 Kb/cM. This information will be useful for further research efforts, including gene cloning and genome structural analyses.

### Quantitative Trait Locus Identification

Mapping QTLs in jujube is challenging, because jujube is a self-incompatible plant with high heterozygosity and a long growth and breeding cycle. Therefore, it is difficult to generate a population fitting for QTL mapping, such as F_2_ and recombinant inbred lines. The number of samples for a crossed population is also smaller than annual crops. With the development of sequencing and its application in marker screening, high-resolution linkage maps have been successfully employed for QTL fine mapping (Chapman et al., 2012). Previously, no studies have included mapping and QTL identification using the F_1_ population of “Dongzao” × “Jinsi 4.” Shen (2005) first conducted a QTL study for leaf traits using AFLP markers, detecting 25 QTLs for leaf traits (leaf length, leaf width, leaf length/width, leaf area), explaining 8.2% to 34.9% of the variance. In 2009, Qi et al. (2009) identified nine QTLs in the same population using AFLP and RAPD markers for agronomic traits of needling (long needle on trunk, short needle on trunk, long needle on branch, and short needle on branch), explaining 8.2% to 44.2% of the variance. The genetic maps these studies constructed were of lower resolution and unsaturated, and possibly unable to reliably capture QTLs.

In contrast, markers in our constructed map spanned 2,167.54 cM with a shorter average marker interval of 0.358 cM, which would facilitate the accurate localization of QTLs. A total of 30 QTLs for seven traits and for the first time, for some important jujube leaf-related traits, such as specific leaf weight and chlorophyll content, have been reliably mapped. These markers are easily located in the corresponding genome sequences and can be used to study candidate genes related to traits located on those chromosomal regions. Therefore, our findings will enhance the efficiency of future gene discovery studies in jujube.

In this study, only QTLs associated with leaf length were located in the same linkage group as those in previous studies, although at different positions. The QTLs affecting leaf length were found on chromosomes LG1 and LG11 both in our study and in previous studies (Shen, 2005). However, there were additional novel QTLs detected in our study that were not previously identified. Our study also mapped QTLs for chlorophyll content for the first time in Chinese jujube. Interestingly, all of these were located on chromosome LG5. The three QTLs affecting needling were also located on chromosome LG5, which was distinct from previous studies (Qi et al., 2009). The 30 QTLs that showed stable and significant effects for phenotype of leaf and needling traits would be valuable resources for candidate gene exploration in the near future. Combined with the whole genome sequence of jujube, genes surrounding these QTLs could be investigated as candidate genes for further screening and verification.

## Conclusions

In this study, a high-density genetic linkage map of Chinese jujube was constructed using GBS. This linkage map contained 12 linkage groups with a low inter-marker distance of 0.358 cM. A total of 27 QTLs associated with leaf traits, and 3 QTLs associated with needling traits were identified. The findings would be helpful in marker-assisted selection studies for jujube. The high-density linkage map will also serve as a foundation for jujube genetic improvement.

## Data Availability Statement

The SNP datasets generated for this study can be found in the Dryad repository (doi: 10.5061/dryad.np574cj).

## Author Contributions

ZW and XL designed the whole research program. ZW performed the whole experiments, analyses, and manuscript preparation. ZZ, HT, and QZ worked on and improved the original draft and figures. The manuscript was approved by all co-authors.

## Funding

This study was financially supported by National Basic R & D Program of China (2018YFD1000607), the Natural Science Fund of Shandong Province (ZR2017PC018) and Shandong Provincial Key R & D Program (2018GNC11306).

## Conflict of Interest

The authors declare that the research was conducted in the absence of any commercial or financial relationships that could be construed as a potential conflict of interest.
