# Supplementary material for: High-Density Genetic Map Construction and QTL Mapping of Leaf and Needling Traits in Ziziphus jujuba Mill
Source: Front Plant Sci. 2019 Nov 22;10:1424. doi: 10.3389/fpls.2019.01424 (PMC6882864; doi:10.3389/fpls.2019.01424)
Supplement: Supplementary file 9 [file Presentation_1.zip › Image 3.pdf]

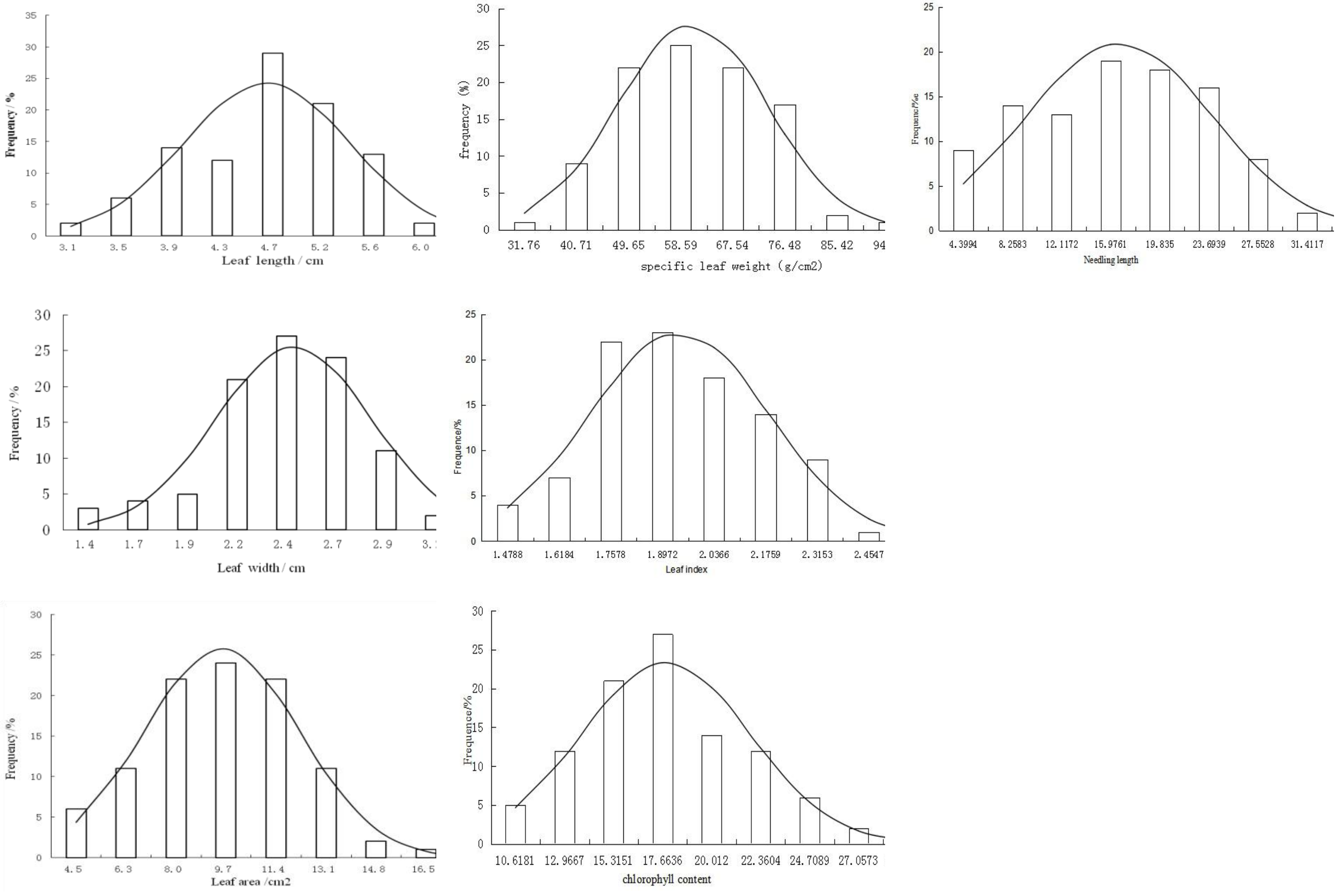


Supplementary figure S3. Segregation of leaf length,leaf width,leaf area,leaf index,specific leaf weight,chlorophyll content and needling length traits in F1 population
