# Supplementary figures and images for: High-Density Genetic Map Construction and QTL Mapping of Leaf and Needling Traits in Ziziphus jujuba Mill
Source: Front Plant Sci. 2019 Nov 22;10:1424. doi: 10.3389/fpls.2019.01424 (PMC6882864; doi:10.3389/fpls.2019.01424)

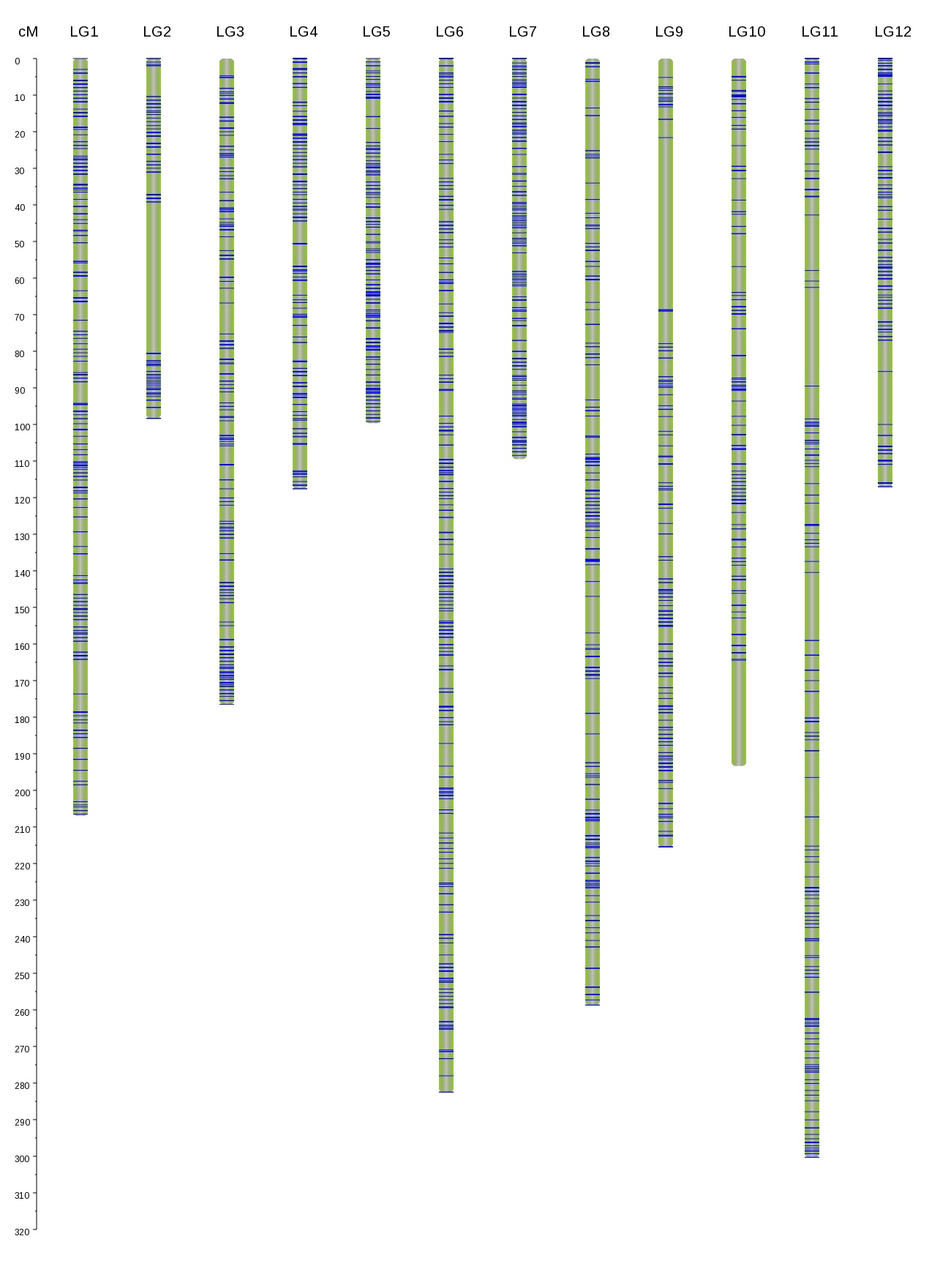

Supplement: Supplementary file 9 [file Presentation_1.zip › Supplementary Figure S1 Male genetic map with 12 linkage groups. The x axis indicates the numbers of linkage groups_ the y axis indicates the genetic length (cM)..jpg]

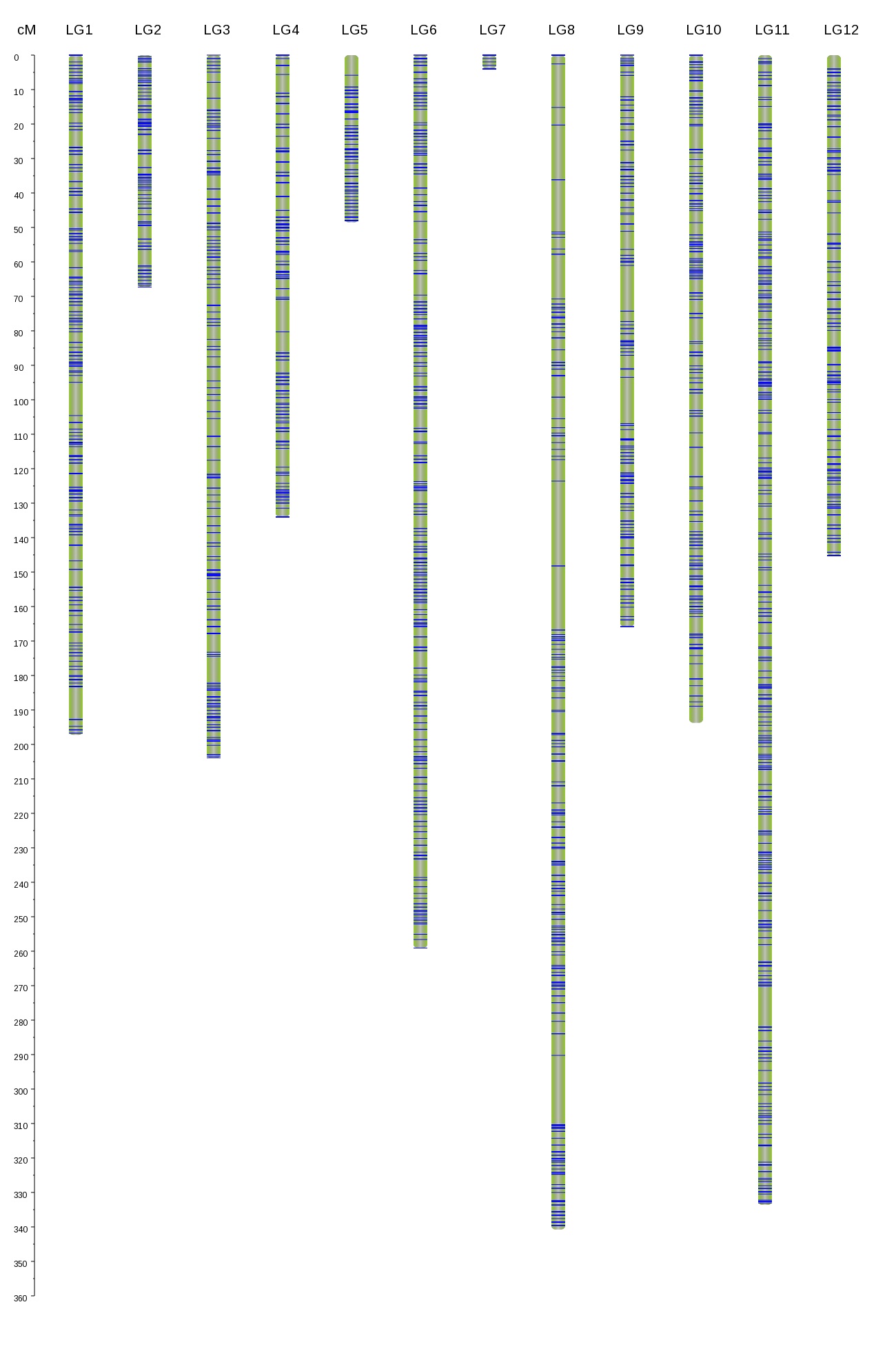

Supplement: Supplementary file 9 [file Presentation_1.zip › Supplementry Figure S2 Famle genetic map with 12 linkage groups. The x axis indicates the numbers of linkage groups_ the y axis indicates the genetic length (cM)..jpg]
